# Editorial: Pesticides exposure and public health

**DOI:** 10.3389/fpubh.2023.1211115

**Published:** 2023-07-04

**Authors:** Sahar T. Issa, Aseel A. Takshe, Nisreen H. Alwan, Iffat ElBarazi

**Affiliations:** ^1^Department of Public Health, Canadian University Dubai, Dubai, United Arab Emirates; ^2^Environmental and Public Health Department, Abu Dhabi University, Abu Dhabi, United Arab Emirates; ^3^College of Medicine and Health Sciences, Institute of Public Health, United Arab Emirates University, Al Ain, United Arab Emirates

**Keywords:** environmental health, pesticides, exposure, risk assessment, biological monitoring

The rapid increase in the world population has resulted in boosting the demand for food supply and agricultural food products coupled with an intensification of pesticides applications. At the global level, pesticides applications have increased by more than 50% between 1990 and 2010 (1). The total pesticides trade was estimated to be around 5.9 million tons in 2019, which accounts for around 35.5 billion US dollars (1). Thus, pesticides use is expected to double in the next 10 years in developing countries (2). This increase will raise the alarm over the dangers of pesticides and there will be a need to address pesticide safety in developing countries.

The widespread use of pesticides in agricultural and domestic settings is a serious threat to the environment and public health. The effects of excessive and inappropriate uses of pesticides on the environment and human health are recognized worldwide (1–4). Due to their persistent nature, pesticides remain in the environment for a long period and gradually enter food systems (4). Exposure to pesticides occur via different environmental media such as air, water, soil, and food and can result in both acute and chronic effects. Acute toxic effects follow poisoning by pesticides and include nausea, headaches, dizziness, skin and eye irritation, muscle paralysis, and respiratory failure. Chronic health effects include allergic reactions, impaired immune, endocrine, and reproductive functions, liver damage, peripheral neuropathies, neurobehavioral disorders, and cancers (2, 4, 5). Every year, more than three million poisoning cases, 220,000 deaths, and 750,000 chronic illnesses are caused by pesticides use, moslty in developing countries (2).

The current state of environmental degradation caused by pesticides and the associated health effects are not well understood given the diversity, complex nature, toxicity, bioaccumulation, mobility, and persistence of pesticides in the environment (1, 5). Thus, there is an urgent need to understand the occurrence and fate of pesticides in the environment to explore the health effects associated with exposure to pesticides and monitor and evaluate such risks. This will aid in finding sustainable and innovative strategies for pesticides management and remediation and informing policymakers on the most appropriate approaches to minimize pesticide exposure and their adverse health effects.

This Research Topic highlights the recent developments in the field of pesticides and provides evidence on the effects of pesticides exposure on human health. Our Research Topic includes two original research studies and two systematic reviews that explore the mechanisms of action of pesticides and associated health effects, biological monitoring and risk assessment of exposure to pesticides residues, and possible approaches and remedial measures for improved and safer use of pesticides. The original research studies highlight pesticides applications in the occupational setting in Ethiopia and China. The systematic reviews focus on the health effects of pesticides and the importance of validated analytical methods to detect and quantify pesticides residues in food.

## Author contributions

SI has drafted the editorial. AT, NA, and IE have revised it for important intellectual content. All authors contributed to the article and approved the submitted version.
